# Supplementary material for: Modulation of Gut Microbial Community and Metabolism by Bacillus licheniformis HD173 Promotes the Growth of Nursery Piglets Model
Source: Nutrients. 2024 May 15;16(10):1497. doi: 10.3390/nu16101497 (PMC11124511; doi:10.3390/nu16101497)
Supplement: Supplementary file 1 [file nutrients-16-01497-s001.zip › nutrients-2962734-supplementary.pdf]

Table S1 Composition and nutrient levels of the basal diet(air-dry basis)

Items	Content
Ingredients	
Corn	60%
Wheat	10%
barley	5%
Soybean meal	18%
Fermented soybean meal	3%
Soybean oil	0.50%
Premix	4%
Total	100%
Nutrient levels	
Digestible Energy /（MJ/Kg）	10.04
Crude Protein	16.50%
Digestive lysine	1.10%

Table S2 Body weight and ADG of three groups in nursery piglets

Item	Control	0.02% BL	0.04% BL
Body weight at 0d (kg)	8.7 ± 0.15	8.8 ± 0.13	8.8 ± 0.12
Body weight at 14d(kg)	13.1 ± 0.20	13.7 ± 0.19	13.6 ± 0.24
Body weight at 28d(kg)	17.7 ± 0.33	18.9 ± 0.31	18.5 ± 0.40
0d to 14d ADG (g/day)	311.90 ± 5.14	349.21 ± 7.51	343.25 ± 10.60
15d to 28d ADG (g/day)	328.17 ± 10.87	373.81 ± 11.43	348.41 ± 12.25
0d to 28d ADG (g/day)	320.04 ± 7.90	361.51 ± 9.16	345.83 ± 11.32

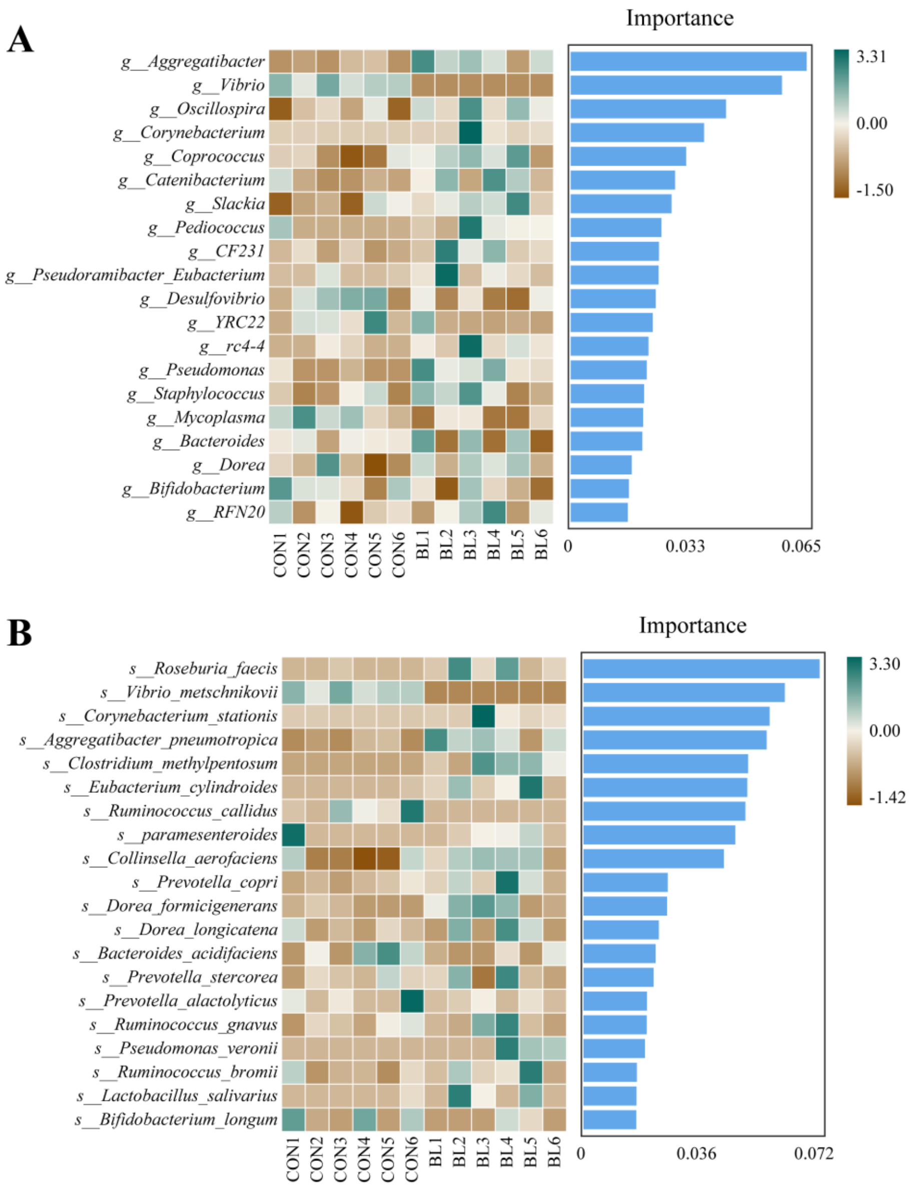

Figure S1. Random forest analysis of the relative abundance at the genus and species level. (**A**) the 20 genera with the highest level of importance; (**B**) the 20 species with the highest level of importance. n = 6.

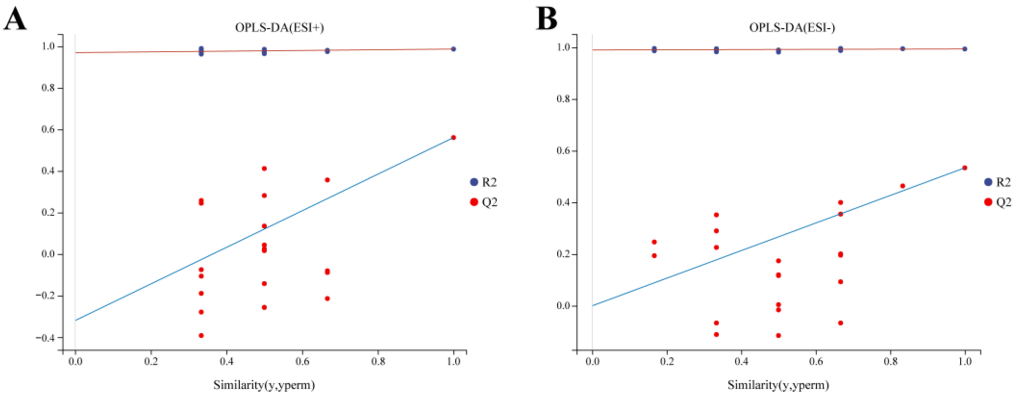

Figure S2. Permutation test of the OPLS-DA model. (**A**)ESI+; (**B**)ESI-. n = 6.
